# Characterization of Fossil and Renewable Fuels

**DOI:** 10.1155/2018/5264942

**Published:** 2018-01-01

**Authors:** Xing Fan, Bo Chen, Xun Hu, Nilusha Sudasinghe, Ya-He Zhang

**Affiliations:** ^1^Department of Applied Chemistry, China University of Mining and Technology, Xuzhou 221116, China; ^2^Department of Chemical & Petroleum Engineering, University of Wyoming, Laramie, WY 82071, USA; ^3^Fuels and Energy Technology Institute, Curtin University, Bentley, WA 6102, Australia; ^4^Bioscience Division, Los Alamos National Laboratory, Los Alamos, NM 87545, USA; ^5^State Key Laboratory of Heavy Oil Processing, China University of Petroleum, Beijing 102249, China

The special issue aims to provide contributions from a variety of topics relating to the characterization of properties, structures or molecular compositions of fossil fuels, and biomass, as well as the corresponding derivatives or products through thermal, chemical, and biochemical methods.

The special issue presents six papers relating to biomass, crude oil, and coal. We feel that the published articles represent a certain wide range of researches in the scope of special issue. A series of analytical techniques in chromatography, microscopy, and spectroscopy were included in the researches. This special issue is dedicated to the readers in the research fields of analytical chemistry, biochemical engineering, chemical engineering, geology, and mineral processing engineering.

In X. Yue et al.'s paper, liquefaction residue of a bituminous coal was subject to hydroconversion reactions and the products were analyzed using Fourier transform infrared spectroscopy and gas chromatography/mass spectrometry. More than 200 organic compounds were detected in the products and divided into alkanes, aromatic hydrocarbons, phenols, ketones, ethers, and other species.

Scanning electron microscopy was applied in Z. Lu et al.'s research to characterize particle morphology, including size and shape, which is an important factor significantly influencing the physical and chemical properties of biomass material. An image segmentation algorithm based on particle geometrical information was proposed to recognize the finer clustered powders.

Biomarkers derived from early living organisms play an important role in oil and gas geochemistry and exploration since they can record the diagenetic evolution of the parent materials of crude oil and reflect the organic geochemical characteristics of crude oil and source rocks. Therefore, gas chromatography/mass spectrometry was applied by X. Zhang et al. to study the biomarker compounds of crude oil in Southwestern Yishan Slope of Ordos Basin.

S. Wang et al. used X-ray fluorescence spectrometer, X-ray diffractometer, X-ray photoelectron spectrometer, and field emission electron probe microanalyzer to study the composition of heavy products in fine coal powders separated by ultrasonic vibration gas-solid fluidized bed. The particle separation process based on density was strengthened by the introduction of ultrasonic vibration.

The paper by Y.-C. Lu et al. reported componential characterization of extractable species in wheat straw. Analytical methods like Fourier transformed infrared spectroscopy, gas chromatography/mass spectrometry, X-ray photoelectron spectroscopy, transmission electron microscopy, energy dispersive spectrometry, and electron probe microanalysis were used, and detailed molecular information was provided.

Analytical strategies involved in the componential characterization of bio-oil produced from lignocellulosic biomass were reviewed by Y. Lu et al. The use of chromatographic and spectrometric methods such as gas chromatography/mass spectrometry and high performance liquid chromatography/mass spectrometry was highlighted. Fourier transform infrared spectroscopy and nuclear magnetic resonance were also mentioned.

*Xing Fan* *Bo Chen* *Xun Hu* *Nilusha Sudasinghe* *Ya-He Zhang*

